# Cost‐effectiveness of root canal treatment compared with tooth extraction in a Swedish Public Dental Service: A prospective controlled cohort study

**DOI:** 10.1002/cre2.759

**Published:** 2023-06-29

**Authors:** Emma Wigsten, Thomas Kvist, Magnus Husberg, EndoReCo, Thomas Davidson

**Affiliations:** ^1^ Department of Endodontology, Institute of Odontology, The Sahlgrenska Academy University of Gothenburg Gothenburg Sweden; ^2^ Public Dental Service Region Västra Götaland Gothenburg Sweden; ^3^ Department of Health, Medicine and Caring Sciences Linköping University Linköping Sweden

**Keywords:** cost‐effectiveness analysis, EQ‐5D‐5L, patient‐reported outcome measures, quality‐adjusted life years

## Abstract

**Objectives:**

To evaluate the cost‐effectiveness of root canal treatment (RCT) compared with a tooth extraction in a general dental practice setting, with reference to cost per quality‐adjusted life‐year (QALY) gained over 1 year.

**Material and Methods:**

This is a prospective controlled cohort study based on patients either starting RCT or undergoing extraction at one of six Public Dental Service clinics in the county of Västra Götaland, Sweden. From a total of 65 patients, 2 comparable groups were formed: 37 started RCT and 28 underwent extraction. A societal perspective was used for the cost calculations. QALYs were estimated, based on the EQ‐5D‐5L given to the patients at their first treatment appointment and then after 1, 6, and 12 months.

**Results:**

The total mean cost of RCT ($689.1) was higher than for extraction ($280.1). For those patients whose extracted tooth was replaced, the costs were even higher ($1245.5). There were no significant intergroup differences in QALYs, but a significant improvement in health state values in the tooth‐preserving group.

**Conclusions:**

In the short term, extraction was cost‐effective compared with preserving a tooth with RCT. However, the potential need for future replacement of the extracted tooth, by an implant, fixed prosthesis, or removable partial dentures, may change the calculation in favor of RCT.

## 1 INTRODUCTION

Root canal treatment (RCT) is often initiated to relieve symptoms such as pain or tenderness (Bjørndal et al., 2006; Wigsten et al., 2019). The short term aim is therefore to relieve the symptoms and in the longer term to preserve the root‐filled tooth as a functional unit with healthy periradicular tissues. Studies have also shown that undergoing RCT can improve the patient's quality of life (QoL) (Diniz‐de‐Figueiredo et al., 2020; Liu et al., 2014). A treatment alternative is tooth extraction, complemented in some cases by prosthetic constructions to replace the lost tooth.

Most clinical prospective follow‐up studies on RCT have been conducted at universities, dental hospitals, or specialist clinics, often with referred patients. Less is known about the effectiveness of endodontic treatment provided by general dental practitioners within the public dental service. The treatment effect can advantageously be studied by using real costs and the patients' perceived QoL, to calculate the cost‐effectiveness by comparing the two treatment alternatives. It may well be that it would be more cost‐effective to extract the affected tooth than to undertake RCT. Dental care resources are limited and the care provided has to be paid for, by the patient, the caregiver, an insurance company, or, through taxes, by society (Zaror & Mariño, 2022). As limited resources must always be prioritized, it is also important to determine whether the treatment has the desired outcome. Cost‐effectiveness studies consider both the effect of treatment and the costs involved and may guide decision‐makers in the prioritization process.

We have previously presented a short term (1 month) nonrandomized study of a cohort of individuals who either started RCT or underwent tooth extraction in a public dental service setting (Wigsten et al., 2020). The results showed that preserving the tooth by RCT achieved a better outcome: in contrast to those who had an extraction, the patients treated by RCT reported improvement in their health‐related QoL (HRQoL). One limitation of the study, however, was the short follow‐up period. A longer follow‐up period would also take into account the following treatment: teeth in which RCT had been initiated would have been completed with a root filling and a permanent coronal restoration and the extracted teeth may have been replaced with different prosthetic constructions.

This study follows the same cohort but for longer. Based on the patients' perceived HRQoL with real‐world data, the two treatment options were compared by cost‐effectiveness analysis. By using the instrument EQ‐5D‐5L (Herdman et al., 2011; The EuroQol group, 1990) it is possible to calculate quality‐adjusted life‐years (QALY). The QALY measure combines the value of a health state with the duration of it (i.e., time). QALYs are common in health economic analyses and enable comparisons with studies of different disorders and interventions (Davidson, 2022; Drummond et al., 2015).

There are a limited number of health economic studies evaluating RCT outcomes and only a few are based on empirical data (Basmadjian‐Charles et al., 2004; Koch et al., 2012): in most studies, cost‐effectiveness has been analyzed primarily by modeling (Kim & Solomon, 2011; Pennington et al., 2009; Schwendicke & Göstemeyer, 2016; Schwendicke & Stolpe, 2015). RCT outcomes are of interest not only to the individual patient and the service provider but also to third‐party stakeholders (Schwendicke & Herbst, 2022). There is therefore a need for further studies which include the health economic aspects. To our knowledge, there are, to date, no published studies in the field of endodontics using health state values or QALYs as the outcome measure. By choosing the start of treatment as the study baseline, it is possible to register the resources required to achieve the goal of a functional root‐filled tooth, such as the number of dental appointments and chairside time, the patient's absence from work, and travel to and from the dental clinic.

The present study was conducted in general dental practice over a period of one year. The aim was to compare the cost‐effectiveness of RCT and tooth extraction, in terms of cost per QALY gained.

## 2 METHODS

### 2.1 Setting

In Sweden, adults usually pay for their own dental care. The current Dental Care Benefits Scheme was introduced in 2008 and provides subsidized care in accordance with the high‐cost protection system (Tandvårds‐ och läkemedelsförmånsverket, 2022a). The Dental and Pharmaceutical Benefits Agency determines which dental procedures are to be subsidized by state tax‐financed funds and how the reference prices are to be calculated (Tandvårds‐ och läkemedelsförmånsverket, 2022b). The reference price then determines how much of the treatment can be compensated. Most dental procedures, including RCT, coronal restoration, and tooth extraction with possible replacement such as implants, are included in the high‐cost protection system. The Dental Care Benefits Scheme is administered by the Swedish Social Insurance Agency (Tandvårds‐ och läkemedelsförmånsverket, 2022b).

### 2.2 Study population

The study population, the baseline characteristics, and 1‐month follow‐up are described in a previous study (Wigsten et al., 2020). In short, the patients were consecutively enrolled for a predetermined period of 8 weeks, either starting RCT or undergoing tooth extraction at one of six public dental clinics in the county of Västra Götaland, Sweden (Supporting Information).

All treatments were preceded by a diagnosis followed by a treatment plan determined by the general dental practitioner in dialog with the patient. Sixty‐five patients met the inclusion criteria, which were: (i) RCT was started or a tooth was extracted; (ii) the patient was over 18 years of age; (iii) could read the Swedish language; and (iv) was capable of giving voluntary informed consent. The number of patients who declined to participate or were ineligible to participate due to language difficulties, or physical or mental illnesses, was registered and the planned treatment was noted. The recruitment period lasted from August to December 2017. All clinics were affiliated with the Swedish Social Insurance Agency.

### 2.3 The questionnaire

The initial questionnaire was distributed at the first treatment appointment, representing the study baseline. It was distributed after the set treatment plan and was responded to either in the treatment room or the waiting room. Subsequent questionnaires were sent by post, 1, 6, and 12 months after baseline, respectively. To increase the response rate, a reminder was sent 3 weeks later, with an additional telephone call if the questionnaire was not returned. The mailings took place between September 2017 and December 2018. All material was in Swedish.

The questionnaire contained the instrument EQ‐5D‐5L and five additional questions with personal demographic data. The instrument was used with permission of the EuroQol Group (EQ‐5D). The EQ‐5D‐5L has two sections: a descriptive system with five statements representing five dimensions of health, each with five response levels, and a visual analog scale (EQ‐VAS) (Herdman et al., 2011; The EuroQol group, 1990). A numerical description of the HRQoL is then generated, which can be equipped with predefined values, usually based on different populations (Burström et al., 2020; Devlin et al., 2018; Dolan, 1997; van Hout et al., 2012). These values can also be used to create QALYs when multiplied with time. For the analysis, the tariffs 3 L (crosswalk to the Dolan tariff) (Dolan, 1997; van Hout et al., 2012) and 5 L (Devlin et al., 2018) tariffs representing the population in the United Kingdom were used, as well as a tariff representing experienced‐based values in Sweden (Burström et al., 2020). The patient's general health is then rated on a (EQ‐)VAS, ranging from “worst imaginable health state” (Score 0) to “best imaginable health state” (Score 100) (Herdman et al., 2011; The EuroQol group, 1990).

The patients' demographic data included main occupation (employee/self‐employed, senior citizen, or other), the highest level of education (elementary school, high school, university, or other, more or less than 2 years), whether leave from paid work was taken to keep the dental appointment, what means of transport was used to attend the dental clinic, whether the appointment involved additional costs such as paid parking, possible sick leave, and so on.

Absent data were designated in the analysis as missing. All five dimensions had to have valid statements, otherwise, the data were treated as missing and excluded from the statistical analysis with the tariffs. If there was a discrepancy between the numbers in the box and the “X” on the EQ‐VAS, then the numbers in the box were assumed to be valid. The number of days between the first appointment and answering the questionnaires was also registered.

### 2.4 The dental records

All teeth were monitored through the computerized dental records for 1 year following the first appointment. The data were handled anonymously by the previous allocation of unique identification numbers in the Excel data sheet. When data were missing, the data sheet cell was left blank and designated in the analysis as missing.

For the teeth in which RCT was initiated, completed treatment was registered as “root‐filled” or “extracted”. The remainder were registered as “not completed”. The permanent coronal restoration was classified as “direct” (i.e., direct resin composite or glass ionomer filling) or “indirect” (i.e., tooth‐supported crown fabricated by a dental technician). Cases where no direct or indirect restoration was to be found were registered as “unspecified”. Posts were classified as directly or indirectly fabricated. For the extracted teeth, the main reason for extraction and type of replacement during follow‐up were registered.

The total number of appointments and the chairside time (i.e., hours) were registered, as was the time interval, in days, between treatment start and completion.

### 2.5 Costs and cost‐effectiveness

The CHEERS checklist was followed (Husereau et al., 2022). The material was analyzed from a societal perspective. The incremental cost‐effectiveness ratio was presented as cost per QALY gained. All costs are calculated in Swedish krona (SEK) and converted to USD according to the exchange rate prevailing on September 30, 2022 (1 SEK = $0.09). As the duration of the study was limited to 1 year only, no discounting was included.

All dental fees charged during the year were registered. Each dental treatment has a specific three‐digit code, which then determines the direct patient fee and the subsidy from the Swedish Social Insurance Agency. The fees charged are presented in three different ways: (i) the total fees set by the county of Västra Götaland, (ii) the total fees representing the national reference price, and (iii) necessary chairside time compared with set hourly income (i.e., time multiplied by hourly income). The reason for examining hourly income (iii) was that the fees charged for dental care are intended to cover all the clinics' costs such as premises, dental materials, equipment, salaries, consultations, etc. However, dental technicians' labor and materials are not included in the hourly income. In 2017 and 2018, the set hourly income was $179 in general dental clinics and $233 in specialist clinics.

For indirect costs, the productivity loss is calculated at an hourly rate of $26.9, approximately the cost an employer has, including payroll taxes. A standard calculation was made for those who stated that they traveled by car ($1.7/10 kilometers, calculated 40 km round trip). Finally, the costs of parking tickets and travel by public transport are included as indicated in the questionnaires.

### 2.6 Statistical analyses

IBM SPSS Statistics 25 and Excel 2016 (Microsoft Corp.) were used for statistical calculations. The intention‐to‐treat was applied. The categoric variables are presented as numbers and percentages, and for the continuous variables, the distribution is expressed as the mean, SD, median, and range (minimum–maximum). The statistical uncertainty is described as a 95% confidence interval and/or *p*. The independent samples *t* test was used to test the mean differences between groups (independent). The difference in mean between the start and 12 months was tested with a paired sample *t* test. In the calculation of health states, carry forward–backward was used to manage missing responses. Distribution was presented as a percentage (*n*) and differences were tested by *χ* ^2^. A *p* < .05 was defined as a statistically significant difference.

### 2.7 Ethical considerations

The study protocol was approved by the regional ethical committee in Gothenburg, Sweden, in 2016 (dnr: 817‐16). The study was outlined according to the STROBE checklist and statements. Written informed consent was obtained from all the participating patients.

## 3 RESULTS

Sixty‐five patients were included: 34 (52.3%) men and 31 (47.7%) women, with a mean age of 55.5 years (SD = 15.1). Thirty‐seven (56.9%) patients started RCT and 28 (43.1%) underwent extraction. The respective response proportions were 95.4% (*n* = 62), 76.9% (*n* = 50), 75.4% (*n* = 49), and 83.1% (*n* = 54). Forty patients answered all four questionnaires (61.5%). The molars dominated (*n* = 42, 64.6%). The patients' demographic data at baseline are presented in Table 1.

**Table 1:** Baseline registrations and demographic data for 65 patients treated in a general public dental service.

	RCT	Extraction	*p*
Variable/Category	(*n* = 37)	(*n* = 28)	
Main occupation			
Employee/self‐employed	24 (70.6)	17 (60.7)	
Senior citizen	8 (23.5)	8 (28.6)	
Other (student, unemployed, on sick leave, etc.)	2 (5.9)	3 (10.7)	.663
Level of education			
Elementary school	5 (14.7)	7 (25.0)	
High school	19 (55.9)	15 (53.6)	
University or other <2 years	5 (14.7)	1 (3.6)	
University or other >2 years	5 (14.7)	5 (17.9)	.405
Loss of income due to dental attendance^a^			
Yes	12 (36.4)	11 (39.3)	
No	21 (63.6)	17 (60.7)	.814
Time (in hours)^b^ Mean (SD)	2.7 (2.0)	2.7 (1.9)	.940
Median (range)	2.0 (1.5‐8.0)	2.0 (1.0‐8.0)	.680
Means of transport to the clinic			
Public transport	4 (11.8)	1 (3.6)	
Car	26 (76.5)	20 (71.4)	
Walking, cycling, and so on	4 (11.8)	7 (25.0)	.241
Extra cost due to attendance			
Yes	5 (14.7)	0 (0.0)	
No	29 (85.3)	28 (100.0)	.034

*Note*: Values presented as *n* (%).

Abbreviation: RCT, root canal treatment.

^a^ The following data were missing: “Loss of income due to dental attendance”: one case.

^b^ Twelve versus 6 patients, respectively, registered time due to dental attendance.

### 3.1 Teeth in which RCT was started

Most of the teeth were root‐filled within 1 year (*n* = 27, 73.0%), the remainder were either not completed (*n* = 2, 5.4%) or had been extracted (*n* = 8, 21.6%). One tooth was referred and completed at the specialist endodontic clinic. Most teeth were restored by direct restoration (*n* = 17, 63.0%), others by indirect restoration (*n* = 7, 25.9%), or were still unspecified (*n* = 3, 11.1%). It took an average of 3.7 appointments (SD = 1.5), 3.4 h (SD = 1.6), and a time interval of 119 days (SD = 94.5) to achieve one functional root‐filled tooth, that is, from the initial treatment appointment until the treatment was considered completed, that is, root‐filled with subsequent coronal restoration. Five teeth were restored with a post, directly (*n* = 1) or indirectly fabricated (*n* = 4).

For most of the teeth in which RCT was started but then subsequently extracted (*n* = 8), an endodontic‐related indication was registered (*n* = 5, 62.5%), for example, apical periodontitis or a dentinal crack. In other cases, the tooth was extracted at the request of the patient (*n* = 2) or because of marginal periodontitis (*n* = 1). Three of the extracted teeth in this group were replaced with a prosthetic construction (37.5%; *n* = 1, removable partial denture; *n* = 2, tooth‐supported bridges). None of these was a molar. One root‐filled tooth was extracted 153 days after the completed root filling and subsequent coronal restoration. The reason was a vertical root fracture.

At the end of the follow‐up, all teeth in which RCT was initiated required a mean of 3.6 appointments (SD = 1.5), corresponding to a mean of 3.2 h (SD = 1.5) (Table 2).

**Table 2:** Total number of appointments (scheduled and unscheduled) and chairside time (hours) required for all 65 teeth during a year.

Variable/Category	Appointments	Chairside time
RCT (*n* = 37)		
Root‐filled (*n* = 27)	99	91.3
Mean (SD)	3.7 (1.5)	3.4 (1.6)
Median (range)	4.0 (1.0–7.0)	3.3 (1.0–6.8)
Not completed (*n* = 10)	33	27.2
Mean (SD)	3.3 (1.3)	2.7 (1.2)
Median (range)	4.0 (1.0–5.0)	3.2 (0.7–3.8)
Total	132	118.5
Mean (SD)	3.6 (1.5)	3.2 (1.5)
Median (range)	4.0 (1.0–7.0)	3.3 (0.7–6.8)
Tooth extraction (*n* = 28)		
Extraction without replacement (*n* = 25)	25	23.9
Mean (SD)	1.0 (0.0)	1.0 (0.4)
Median (range)	1.0 (1.0–1.0)	0.8 (0.5–2.0)
Extraction with replacement (*n* = 3)	13	12.5
Mean (SD)	4.3 (0.6)	4.2 (0.6)
Median (range)	4.0 (4.0–5.0)	4.0 (3.7–4.8)
Total	38	36.4
Mean (SD)	1.4 (1.1)	1.3 (1.1)
Median (range)	1.0 (1.0–5.0)	0.8 (0.5–4.8)
All teeth (*n* = 65)	170	154.9
Mean (SD)	2.6 (1.7)	2.4 (1.6)
Median (range)	2.0 (1.0–7.0)	1.9 (0.5–6.8)

Abbreviation: RCT, root canal treatment.

### 3.2 Teeth that were extracted

Eight teeth (28.6%) were previously root‐filled. The reasons given for extraction were in most cases an endodontic‐related complication (*n* = 14, 50.0%), for example, apical periodontitis, perforation, or a dentinal crack. Nine teeth (32.1%) were extracted due to tooth substance loss. In other cases, the cause was stated to be at the request of the patient (*n* = 3) or marginal periodontitis (*n* = 1). In one case, no reason was recorded (*n* = 1).

Three of the extracted teeth in this group were replaced with a prosthetic construction within the year (10.7%; *n* = 1, removable partial denture; *n* = 2, tooth‐supported bridges). To achieve one functional tooth, that is, from the initial treatment appointment to completion of treatment required an average of 4.3 appointments (SD = 0.6), 4.2 h (SD = 0.6), and a time interval of 189.0 days (SD = 110.4). No molar was replaced with a prosthetic construction.

All teeth that were extracted within 1 year required a mean of 1.4 appointments (SD = 1.1), corresponding to a mean of 1.3 h (SD = 1.1) (Table 2).

### 3.3 Costs and cost‐effectiveness

For all three types of estimate (i–iii), RCT with subsequent restoration incurred a higher total direct cost than extraction (Table 3). However, the highest cost was for patients whose extracted tooth was replaced within a year. The results for the three different types of cost estimates were similar.

**Table 3:** Cost calculations.

	Charged fees	Reference prices	Hourly costs
RCT (*n* = 37)			
Root‐filled (*n* = 27)	748.9 (377.7)	730.1 (324.2)	612.7 (284.2)
Not completed (*n* = 10)	394.3 (401.0)	368.1 (345.7)	487.3 (207.6)
Mean (*n* = 37)	653.1 (410.7)	632.3 (363.8)	578.8 (268.9)
Extraction (*n* = 28)			
Without replacement (*n* = 25)	130.0 (40.5)	129.5 (43.5)	171.6 (63.9)
With replacement (*n* = 3)	1245.5 (148.6)	1099.0 (50.5)	747.4 (107.8)
Mean (*n* = 28)	249.5 (355.7)	233.4 (308.4)	233.3 (193.3)
All teeth (*n* = 65)	479.2 (434.6)	460.5 (392.7)	430.0 (293.6)

*Note*: All costs are presented in USD and values presented as Mean (SD).

Abbreviation: RCT, root canal treatment.

Twelve patients (36.4%) in the RCT group reported a loss of income at the first appointment (mean = 2.7 h, SD = 2.0). In the extraction group, 11 patients (39.3%) reported a loss of income (mean = 2.7 h, SD = 1.9; *p* = .81). If an hour is valued at $26.9, this corresponds to a mean value of $26.5 for the tooth‐preserving group and $28.5 for the extraction group.

Most patients drove to and from the clinic by their own car (*n* = 46, 74.2%). The others traveled by bus, cycled, or walked. There was no statistically significant difference between the treatment groups (*p* = .65). On average, 3.6 appointments were required for the tooth‐preserving group (Table 2), which would render an additional mean cost of $8.4 for transport. In addition, extra costs (*n* = 12) were registered in the form of parking tickets, public transport, and so on, to a mean total cost of $1.3. In the extraction group, 1.4 appointments were needed, which would imply an additional cost of $3.3 for traveling by car. Extra costs (*n* = 4) were registered in the form of parking tickets, public transport, and so on., to a mean total cost of $0.9.

**Figure 1:**
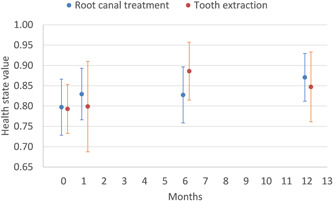
The mean health state values, estimated by the EQ‐5D‐5L using the crosswalk to the Dolan tariff (Dolan, 1997; van Hout et al., 2012) comparing root canal treatment and tooth extraction from baseline to 12 months on, presented with 95% confidence interval. For the tooth‐preserving group, the number of respondents was at baseline: 31, 1 month: 29, 6 months: 27, and 12 months follow‐up: 29. For the extraction group, the number of respondents was at baseline: 26, 1 month: 20, 6 months: 20, and 12 months follow‐up: 21.

The health state values, using the crosswalk to the Dolan tariff (Dolan, 1997; van Hout et al., 2012), provided similar scores for the two groups on all four occasions (Figure 1). However, there was a tendency toward a lower health state value in the tooth‐preserving group after 6 months, but higher at 12 months. When evaluating all responses, the values increased from 0.80 to 0.87 (*p* = .015) between baseline and 1‐year follow‐up with RCT, whereas the value of tooth extraction changed from 0.79 to 0.85 (*p* = .060) during the same period. Both treatment options showed a tendency for health state values to increase from baseline to 1‐year follow‐up. There was no statistically significant difference between the groups on any occasion.

For the patients who responded to the questionnaire at baseline and after 12 months, the tooth‐preserving group recorded a significant improvement in health state values from 0.81 to 0.90 (*p* = .012; *n* = 26). A similar improvement could not be seen in the extraction group, which recorded a change from 0.77 to 0.81 (*p* = .297; *n* = 18).

**Figure 2:**
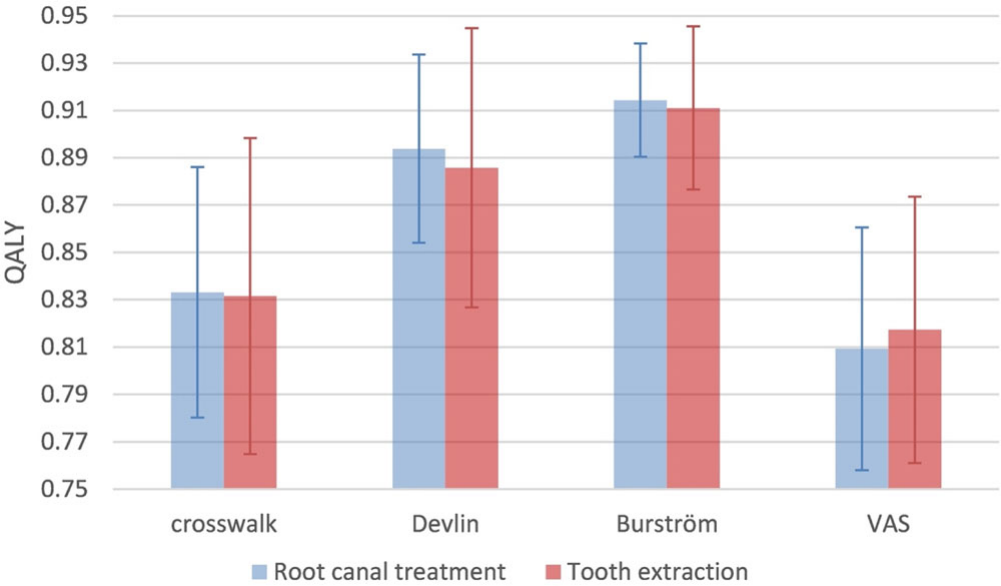
Quality‐adjusted life years (QALYs) during the first 12 months, using different value sets and methods (Burström et al., 2020; Devlin et al., 2018; Dolan, 1997; van Hout et al., 2012) comparing root canal treatment and tooth extraction, presented as the total mean and 95% confidence interval.

When transferring the health state values to QALYs, no differences were found between the treatment alternatives, but different value sets yielded different results (Figure 2). Furthermore, using EQ‐VAS provided the lowest scores. As extraction showed lower total costs with equal QALYs, extraction emerged as the cost‐effective alternative within 1 year of treatment initiation (Table 4).

**Table 4:** Cost‐effectiveness.

	Direct costs	Indirect costs	Total costs	Δ costs	QALY	Δ QALY	ICER
Extraction (*n* = 28)	249.5	30.6	280.1		0.83		
RCT (*n* = 37)	653.1	36.1	689.1	409.0	0.83	0	Dominated

*Note*: All costs are presented in USD (Mean). QALYs at the 1‐year follow‐up are presented (12 months tariff crosswalk).

Abbreviations: ICER, incremental cost‐effectiveness ratio; QALY, quality‐adjusted life year; RCT, root canal treatment.

## 4 DISCUSSION

Two cohorts of patients were followed for 1 year, with one group starting RCT and the other undergoing extraction. The original groups were maintained (intention‐to‐treat) throughout the follow‐up period. With respect to HRQoL, estimated with EQ‐5D‐5L, no significant difference was detected between the treatment options. However, only the patients who underwent the tooth‐preserving treatment registered a significant improvement in perceived HRQoL according to the health states (*p* = .012 and *p* = .015) at the follow‐up. The total mean cost was higher for patients who underwent RCT. Hence, within the context of this study, extraction emerged as the cost‐effective alternative in the short term. However, we acknowledge the high risk of bias since the comparison is not based on a randomized selection of treatment options.

RCT is a common procedure in general dental practice, undertaken primarily to save symptomatic, severely compromised teeth (Wigsten et al., 2019). In Sweden, ~200,000 root fillings are undertaken per year, most in general dental practice (Fransson et al., 2016). There are limited prospective observational cohort studies in general dental practice. In most follow‐up studies, both retrospective and prospective, the baseline is set at the completion of the root filling. By monitoring the teeth from the first appointment, that is, when the RCT was initiated, all the resources required to reach the goal of a functional root‐filled tooth will be recorded. However, despite the resources invested, almost a third of the teeth (27.0%) in this study had not been root‐filled within a year of treatment start. Most such teeth were extracted (80.0%). These results are in agreement with another study from this particular organization (Wigsten et al., 2022). For the general dental practitioner, RCT seems to be a challenging treatment procedure (Dahlström et al., 2017; Wigsten et al., 2022).

The estimation of costs included all interventions to keep a functional tooth at the site (Friedman & Mor, 2004) that is all dental care required to achieve and maintain a root‐filled tooth in function, or alternatively a prosthetic replacement for the extracted tooth. The tooth‐preserving treatment required both more time and more appointments than extraction. However, only three patients in the extraction group underwent replacement of the extracted tooth within a year.

### 4.1 The costs of RCT or extraction

Minor differences were observed between the actual fees charged and the reference price. This suggests that the planned fee can be considered reasonable and adequate to cover the costs of RCT. However, using hourly costs as an estimate ($179/hour) gave a lower cost, which implies that the treatment should yield a financial surplus for this public dental service organization.

In the extraction group, three patients (10.7%) underwent the replacement of their missing teeth. The mean cost for these three patients was $1245.5, which far exceeds the mean cost for maintaining a tooth with RCT ($748.9). The low total number of replaced teeth (*n* = 3) might possibly be explained by the fact that the majority were molars. None of these patients underwent implant treatment.

### 4.2 Health evaluations

To our knowledge, there are no previous studies in the field of endodontics in which EQ‐5D and associated values have been applied. However, the instrument has been used in other areas of dental care, for example, in estimating the costs of treating caries (Kastenbom et al., 2019).

The health state values showed minor differences between the two treatment options on all four occasions. For both treatment options, the health state values generally increased from baseline to the 1‐year follow‐up, where the tooth‐preserving patients registered a statistically significant improvement. At 1 year, the value was highest when using the Dolan tariff (Dolan, 1997) and lowest when using the Swedish tariff by Burström et al. (2020). The EQ‐VAS was slightly lower in comparison with the index values, which means that the patient's own health was valued lower than the general population's perspective on the various health conditions by using different value sets from different countries or regions (Feng et al., 2014).

### 4.3 Cost‐effectiveness

Extraction was the cost‐effective alternative. However, it is uncertain whether extraction would be beneficial in the longer term, as the teeth that were replaced with fixed constructions registered initially higher costs. A longer follow‐up period is required to evaluate whether tooth extraction remains cost‐effective. It is also important to investigate whether the root‐filled teeth require further treatment. It would therefore be of interest to undertake a long‐term clinical and radiographic study of the periapical tissues, postoperative pain, and tooth survival.

The results in this study show that there is no difference in terms of the effect on HRQoL between the two options, but the costs for RCT are significantly higher resulting in extraction as the cost‐effective alternative, in the short perspective. However, as the selection of treatment was done by the patients themselves, the results of the comparison can be questioned. Randomized controlled trials comparing endodontic procedures with extraction and implant are difficult to conduct for several reasons and only a few publications are available; moreover, the conclusions are indecisive (Esposito et al., 2018). Furthermore, the relatively poor outcome of RCT (≈20% extracted before completion) in this particular dental organization (Wigsten et al., 2022) may have influenced patients' estimation of health improvement less favorably. Previous studies have shown that patients are more satisfied with their RCTs if performed by specialists (Dugas et al., 2002; Hamasha & Hatiwsh, 2013). Consequently, the external validity of our findings is uncertain. Therefore, to establish evidence, future studies comparing RCT to alternative treatment options from a cost‐effectiveness perspective should be executed in a variety of settings, and the allocation to different groups should preferably be made random.

## 5 CONCLUSIONS

In this general practice setting, tooth extraction was more cost‐effective in the short term than preserving a tooth by RCT. However, in the long term, the need for future replacement of extracted teeth, by implants, fixed prostheses, or removable partial dentures, may change the balance in favor of RCT.

## AUTHOR CONTRIBUTIONS

**Emma Wigsten**: Conceptualization; methodology; formal analysis; investigation; data curation; writing—original draft; writing—review & editing; visualization; project administration. **Thomas Kvist**: Conceptualization; methodology; writing—original draft; writing—review & editing; visualization. **Magnus Husberg**: Software; formal analysis; writing—original draft; writing—review & editing; visualization. **EndoReCo**: Conceptualization; writing—review & editing. **Thomas Davidson**: Conceptualization; methodology; software; formal analysis; writing—original draft; writing—review & editing; visualization; supervision.

## CONFLICT OF INTEREST STATEMENT

The authors declare no conflict of interest.

## ETHICS STATEMENT

The study protocol was approved by the regional ethical committee in Gothenburg, Sweden, in 2016 (dnr: 817‐16). Verbal and written informed consent was obtained from all the participating patients.

## Supporting information

Supporting Information.

## Data Availability

The data that support the findings of this study are available on request from the corresponding author.
