# Supplementary material for: Cost‐effectiveness of root canal treatment compared with tooth extraction in a Swedish Public Dental Service: A prospective controlled cohort study
Source: Clin Exp Dent Res. 2023 Jun 29;9(4):661–9. doi: 10.1002/cre2.759 (PMC10441609; doi:10.1002/cre2.759)
Supplement: Supplementary file 1 — Supporting Information. [file CRE2-9-661-s001.docx]

|  | 184 patients  started an RCT or  had a tooth extracted | |  | |  |
| --- | --- | --- | --- | --- | --- |
|  | |  | | 68 patients were excluded because voluntary informed consent was not achievable (18 root canal treatments) | |
|  |  |  | |  |  |
|  | |  | | 18 patients declined to participate (7 root canal treatments) | |
|  |  |  | |  |  |
|  | |  | | 13 patients were excluded before the analysis due to erroneous inclusion (*n* = 3), the written consent was missing (*n* = 4), both questionnaires were missing (*n* = 5) and drop-out (*n* = 1) | |
|  |  |  | |  |  |
|  |  |  | |  |  |
|  | |  | | 20 patients were excluded because of extraction of a third molar | |
|  |  |  | |  |  |
|  | |  | |  | |
|  | 65 patients  were included | |  | | |

**Appendix -** Flow chart. Sixty-five patients from 6 public dental clinics were included. Four patients had two teeth treated during the time period: only the first tooth was included (3 extractions and 1 root canal treatment). Patients who underwent extraction of the third molars (n = 20) differed with respect to age and number of remaining teeth. After exclusion of third molars, two relevant and comparable groups were achieved, where the only significant difference was the number of previously root filled teeth (P = 0.03). The two groups comprised a total of 65 individuals: 28 underwent extraction and 37 started root canal treatment.
